# Preparation of a High-Performance Asymmetric Supercapacitor by Recycling Aluminum Paper and Filter Components of Heated Tobacco

**DOI:** 10.3390/ma16196454

**Published:** 2023-09-28

**Authors:** Ha-Yeong Kim, Suk Jekal, Chan-Gyo Kim, Jungchul Noh, Jiwon Kim, Yeon-Ryong Chu, Zambaga Otgonbayar, Won-Chun Oh, Sang Hun Lee, Chang-Min Yoon

**Affiliations:** 1 Department of Chemical and Biological Engineering, Hanbat National University, Daejeon 34158, Republic of Korea; 2 McKetta Department of Chemical Engineering and Texas Material Institute, The University of Texas at Austin, Austin, TX 78712, USA; 3 Department of Advanced Materials Science and Engineering, Hanseo University, Seosan-si 31962, Republic of Korea

**Keywords:** Al paper, tobacco waste, cellulose acetate filter, supercapacitor, electrodeposition

## Abstract

In this study, Al paper and cellulose acetate (CA) filters derived from heated tobacco waste were successfully converted into current collectors and active materials for a supercapacitor device. Typically, heated tobacco contains electrically discontinuous Al paper. First, Al was extracted from the tobacco waste using HCl to produce Lewis acid (AlCl_3_). This acid was then used in an Al electrodeposition process utilizing the chloroaluminate ionic liquid reaction between the acid and the base (RCl) at room temperature. To enhance the conductivity, a supplementary coating of Al metal was applied to the Al paper through electrodeposition, thus re-establishing the electrical continuity of the discontinuous parts and forming an Al-coated current collector. Moreover, the CA filters were carbonized under a nitrogen atmosphere, yielding carbon precursors (C-CA) for the supercapacitor electrodes. To further enhance the electrochemical performance, nickel oxide (NiO) was incorporated into C-CA, resulting in C-CA@NiO with pseudocapacitance. The specific surface area of CA increased with carbonization and the subsequent incorporation of NiO. The as-synthesized C-CA and C-CA@NiO materials were applied to an Al-coated current collector to obtain C-CA- and C-CA@NiO-based electrodes, exhibiting stable electrochemical behavior in the voltage range of −1.0 to 0 V and 0 to 1.0 V, respectively. An asymmetric supercapacitor (ASC) device was assembled with C-CA@NiO and C-CA as the positive and negative electrodes, respectively. This ASC device demonstrated a high specific capacitance of 40.8 F g^−1^, while widening the operating voltage window to 2.0 V. The high electrochemical performance of the device is attributed to the successful Al electrodeposition, which facilitates the electrical conductivity and increased porosity of the C-CA@NiO and C-CA materials. To the best of our knowledge, this is a pioneering study in regards to the conversion of biomass waste into current collectors and active materials to fabricate a practical ASC device. Our findings highlight the potential of reusing Al paper and CA filters from heated tobacco waste as essential components of energy storage devices.

## 1. Introduction

Modern industrial advancements have substantially enhanced the quality of human life. However, reliance on fossil fuels has led to an increase in biomass waste [1]. Biomass waste causes global warming and environmental pollution, in addition to demanding significant disposal costs [2]. One prominent contributor to this waste is tobacco, with an estimated 5.8 trillion tobacco butts discarded annually [3]. Based on consumption methods, tobacco waste can be categorized into conventional and heated tobacco waste. The former results in most components being combusted and lost during smoking [4]. However, for the latter, the heating process retains tobacco waste, thereby generating substantial amounts of biomass waste [5]. Hence, various studies have been conducted in regards to recycling tobacco waste [6,7]. From tobacco waste, Liu et al. fabricated hierarchically porous carbon materials suited for high-performance supercapacitor electrodes [8]. Jekal et al. fabricated a flexible all-solid-state supercapacitor device by fully recycling heated tobacco waste using the polylactic acid gelation method [9]. Tobacco leaves (TL) and cellulose acetate (CA) filters are mainly used as active materials in supercapacitors; however, no study has yet reported the use of Al paper.

Recently, supercapacitors have received considerable attention as promising energy storage devices, owing to the increasing demand for portable electronic devices and hybrid vehicles [10]. Supercapacitors exhibit advantageous characteristics such as rapid charging/discharging capabilities, long-term stability, and exceptional power density [11,12]. Active materials for supercapacitors can be classified into three different types: electric double-layer, pseudo, and hybrid [13]. Electric double-layer types of materials are made up of various carbonaceous materials including carbon nanofibers, activated carbon, and graphene, which store electrical charges through physical absorption–desorption processes at the electrode–electrolyte interface [14,15,16]. In this regard, electric double-layer types of materials exhibit advantages such as high power density and long-term cyclability [17,18,19]. However, the potential applications of electric double-layer types of materials are constrained by their low specific capacitances [20]. Pseudo-types of materials employed in supercapacitors include transition-metal oxides and conducting polymers, which accumulate charges through reversible redox reactions [21]. It is known that the pseudo-type materials can store more energy, thus possessing a higher energy density compared to the electric double-layer types [22]. However, the limitations for pseudo-type materials include poor cycling stability and low electrical conductivity [23]. To overcome the limitations of these two types, hybrid-type materials are synthesized by incorporating metal oxides and conducting polymers into carbonaceous materials [24]. Due to the synergistic effect, hybrid-type materials exhibit various positive characteristics in regards to energy/power density and cyclability [25]. A practical supercapacitor device can be assembled by employing the previously mentioned materials on the positive and negative electrodes. A symmetric supercapacitor device can be constructed by the utilization of identical active materials as positive and negative electrodes. Nevertheless, the application of only one type of material can limit the operational voltage window of the device. On the other hand, asymmetric supercapacitors (ASCs) can be assembled by employing different types of active materials as positive and negative electrodes, attaining widened voltage ranges for practical supercapacitor applications [26,27]. Various synthesis methods and analyses on positive and negative materials for high-performance ASCs have been conducted [28,29]. For instance, Yoon et al. successfully fabricated unique 3D hierarchically structured carbon nanofiber/metal oxide/conducting polymer composites for a high-performance ASC device [24]. Tian et al. synthesized manganese oxide/carbon composites by employing homemade porous carbon and KMnO_4_ for high-energy-density ASC devices [30].

To fabricate hybrid-type materials for supercapacitors, materials can be incorporated using various techniques, including hydrothermal synthesis, electrodeposition, and chemical vapor deposition [31,32,33]. In particular, the hydrothermal method stands out due to its advantages in synthesizing inorganic materials at high temperatures and pressures [34]. Using the hydrothermal method, mixed carbon materials combined with precursor metal ions in an aqueous solution can be efficiently transformed into carbon/metal oxide composite materials [35]. The solvothermal method refers to a method similar to the hydrothermal method, but in this technique, an organic solvent is employed instead of an aqueous solution [36]. While hydro/solvothermal synthesis has its merits, electrodeposition is also a prominent technique for synthesizing carbon/metal oxide hybrid materials [37]. Electrodeposition involves the homogeneous coating of metal oxides onto base materials by applying specific electrochemical conditions, such as constant voltage or current [38]. Noh et al. successfully employed the electrodeposition method to coat MnO_2_ and MoO_3_ onto carbon fibers, which were then assembled into high-performance flexible ASCs [39]. Abbott et al. reported eutectic mixtures of urea and aluminum chloride (AlCl_3_) by utilizing the electrodeposition of aluminum under a layer of decane in a normal humid environment [40]. In addition, the electrodeposition method can be used to coat pure metals to enhance the electrical conductivities of various energy storage device components, including current collectors [41].

The current collector is considered an essential component of supercapacitors, along with the electrode and electrolyte [42]. Depending on its electrical conductivity, the current collector can influence electrochemical performance, cycle stability, and internal resistance [43]. In general, metals such as Al, Ni, and Cu can be employed to fabricate current collectors using electrodeposition and roll-pressing methods [44,45,46]. Current collectors manufactured by electrodeposition exhibit several advantages, including high adhesion, resistance to corrosion, and durability against abrasion [47,48]. In particular, Al is widely utilized in various industrial fields, such as transportation, packaging materials, construction materials, and household appliances, owing to its light weight, high mechanical strength, and superior electrical conductivity [49,50,51]. Al-based current collectors can be fabricated by electrodeposition using metal precursors such as Al(NO_3_)_3_ and AlCl_3_ to supply ions for the formation of Al metals [52,53]. In addition, Al conductive paper included in heated tobacco can be employed as a current collector for supercapacitors using a simple electrodeposition method.

Herein, we present a facile method for preparing current collectors and active materials for supercapacitors, utilizing components from heated tobacco waste, specifically Al paper and CA filters. To our knowledge, this is the first study to report the use of Al paper from tobacco waste as a current collector for energy storage devices. For the Al paper-based current collector, AlCl_3_ powder was extracted from the Al paper and redeposited by electrodeposition to obtain a highly conductive current collector (an Al-coated current collector). Moreover, CA filters and TL were carbonized to obtain the carbon precursors C-CA and C-TL, which served as negative electrodes. In addition, nickel oxide (NiO) was introduced into each carbon precursor to obtain C-CA@NiO and C-TL@NiO, which provided pseudocapacitance to the positive electrodes. Under three-electrode measurements, the C-CA- and C-CA@NiO-based electrodes exhibited specific capacitances of 131.6 and 180.4 F g^−1^ at 1 A g^−1^, respectively. These values are notably superior to those obtained for C-TL- and C-TL@NiO-based electrodes. This increase in capacitance was due to the higher C/O ratio and larger specific surface area of the CA filter, owing to its porous structure. Finally, a practical ASC device was assembled using the Al-coated current collector, with C-CA and C-CA@NiO serving as the active materials. The resulting ASC device exhibited an outstanding specific capacitance of 40.8 F g^−1^ at 1 A g^−1^, widened the operating voltage to 2.0 V, which is sufficiently potent to power a red light-emitting diode (LED) (1.8 V). Thus, this study proposes a viable strategy for the full recycling of heated tobacco waste, leveraging it not only as an active material, but also as a current collector. Such innovative repurposing paves the way for transforming sustainable biomass waste into economic and high-performance supercapacitor devices.

## 2. Materials and Methods

### 2.1. Materials

Heated tobacco butts from HEETS Silver (Phillip Morris Inc., New York, NY, USA) were collected after use. Benzene (99.5%), nickel (II) acetate tetrahydrate (NiAc, 97.0%), hydrochloric acid (HCl, 35.0%), and potassium hydroxide (KOH, 95.0%) were purchased from Samchun Chemical Company (Seoul, Republic of Korea). Gamma-butyrolactone (GBL, 99.0%), poly(vinyl alcohol) (PVA, *M*_w_ of 89,000–98,000), polyvinylidene fluoride (PVDF, *M*_w_ of ~534,000), and 1-methyl-2-pyrrolidinone (NMP, 99.0%) were acquired from Sigma-Aldrich Co. (Burlington, MA, USA). Carbon black was sourced from Tokyo Chemical Industry Co. (Tokyo, Japan). All compounds were used as received, without any additional purification.

### 2.2. Preparation of Aluminum Paper-Based Current Collector

Al paper was obtained from heated tobacco waste and dissolved in an HCl solution (30 mL). Owing to the generation of H_2_ gas during the reaction, the experiment was performed safely in a fume hood. The width, height, and thickness of each Al paper sample were ca. 28, 13, and 0.014 mm, respectively. The amount of Al in the solution was calculated using its density (2.7 g cm^−3^) and molecular weight (26.982 g mol^−1^). After 3 h of complete dissolution, the solution was diluted with deionized (DI) water (800 mL) and vacuum filtered to remove impurities. The filtered AlCl_3_ solution was heated at 100 °C on a hot plate to completely evaporate the moisture content. The resulting AlCl_3_ powder was subsequently placed in a vacuum oven for 12 h at 60 °C. Electrodeposition was performed in a glovebox using the collected AlCl_3_ and GBL. First, GBL was stabilized using a drying process employing CaSO_4_. The electrolyte solution was prepared by mixing GBL and benzene in a volume ratio of 1:6. The as-fabricated AlCl_3_ powder was integrated into this solution to ensure an AlCl_3_ to GBL molar ratio of 1:2.2, and the solution was stirred at room temperature. Subsequently, a three-electrode system was fabricated using Al paper, Pt wire, and Ag/AgCl as the working, counter, and reference electrodes, respectively. Aluminum was deposited on the Al paper at a constant voltage of −2.0 V for 5 min. The product was then dried in an oven at 70 °C for 12 h. Additionally, the surface of the Al-coated paper was deposited with graphite as a protective layer. The graphite layer was prepared by mixing graphite with conductive carbon black and PVDF binder in a mass ratio of 8:1:1 in NMP solvent, maintained under stirring for 12 h. The homogeneous slurry was then applied onto the Al-coated paper using the doctor blade method, and the paper was then dried at 100 °C for 24 h. The as-prepared graphite-coated paper was pressed to 0.45 mm by a roll pressure machine before being employed as the current collector.

### 2.3. Fabrication of C-TL and C-CA Materials

CA filters and TLs were obtained from heated tobacco waste. The CA filters and TLs were washed several times with DI water and immersed in a 6 M KOH solution for 48 h to remove impurities. The samples were then washed with EtOH and DI water before being oven-dried at 80 °C for 12 h. The carbonization of CAs and TLs was carried out in a tubular furnace under a nitrogen atmosphere at 850 °C for 2 h, with a heating rate of 5 °C min^−1^. This process yielded carbonized CA (C-CA) and TL (C-TL).

### 2.4. Synthesis of C-TL@NiO and C-CA@NiO Materials

The C-CA@NiO and C-TL@NiO materials were fabricated by introducing NiO into the C-CA and C-TL materials via a hydro/solvothermal method, using NiAc as a precursor. First, C-CA (0.45 g) was dissolved in isopropanol (20 mL) and stirred for 1 h. Simultaneously, nickel acetate (0.2 g) was dissolved in DI water (5 mL) and stirred for 1 h. The NiAc solution was then gradually added to the C-CA solution, and the mixed solution was placed in a Teflon-lined stainless-steel autoclave. The hydro/solvothermal reaction was performed at 180 °C for 12 h. The resulting C-CA@NiO materials were acquired by washing the resulting solution with isopropanol several times prior to overnight drying in an at 80 °C oven. C-TL@NiO was prepared using a method similar to that used for the fabrication of C-CA@NiO, except that C-TL materials (0.25 g) were used as precursors. The unit of mass loading x (wt%) was evaluated using the following equation [54]:

(1) $$x=\frac{m_{\mathrm{NiO}}}{m_{\mathrm{NiO}}+m_{C}}\times 100\%$$

where $m_{\mathrm{NiO}}$ is the mass of NiO, and $m_{C}$ is the carbonized material (C-CA and C-TL).

### 2.5. Fabrication of Electrodes and the Solid-State Asymmetric Supercapacitor Device

Working electrodes were fabricated by mixing the as-prepared materials, carbon black (as a conducting additive), and PVDF in a mass ratio of 8:1:1, and a small amount of NMP solution was added. The resulting paste was then applied to the Al-coated current collector and dried in an oven (80 °C) for 12 h. In the assembly of the ASC device, C-CA- and C-CA@NiO-based electrodes were employed as negative and positive electrodes, respectively. As a solid electrolyte, PVA (2.0 g) was dissolved in DI water (18 mL) at 80 °C and stirred for 10 h. Subsequently, 1 M Na_2_SO_4_ was mixed with the PVA solution, stirred for 2 h, and maintained at room temperature to obtain the PVA/Na_2_SO_4_ gel-type electrolyte. For the assembly of the ASC device, an optimal gravimetric ratio of C-CA to C-CA@NiO, calculated to be 1.43, ensured the charge balance between the two electrodes; this was determined from the evaluated capacitances in a three-electrode system. The PVA/Na_2_SO_4_ electrolyte was sandwiched between the C-CA- and C-CA@NiO-based electrodes to complete the assembly of the ASC device, which was stored in a fume hood for 24 h to allow the gel-type electrolyte to dry. To lower contact resistance, the outer surface was fixed using polyimide tape and a clip.

### 2.6. Characterization and Electrochemical Measurements

The surface of the Al-coated paper was observed under an optical microscope (BH2-UMA, Olympus, Tokyo, Japan). The morphological structures and elemental compositions (C, O, and Ni) of the CA, TL, C-CA, C-TL, C-CA@NiO, and C-TL@NiO materials were investigated using a field-emission scanning electron microscope (S-4800, Hitachi, Tokyo, Japan) equipped with an energy-dispersive spectrometer (EX-250, HORIBA Ltd., Kyoto, Japan). The porosities of the materials were evaluated based on the N_2_-sorption curves (TriStar II 3020, Micromeritics, Norcross, GA, USA). The crystal structures of the C-CA, C-TL, C-CA@NiO, and C-TL@NiO materials were investigated by X-ray diffraction (D8 Advance, Bruker Co., Billerica, MA, USA) in the 2θ range of 10–80° at 10° min^−1^. The mass losses of the materials were measured using a thermogravimetric analyzer (STA 449 F5 Jupiter, NETZSCH, Selb, Germany) in the temperature range from 40 to 850 °C under N_2_-conditions, with a heating rate of 5 °C min^−1^.

Galvanostatic charge–discharge (GCD) and electrochemical impedance spectroscopy (EIS) analyses were conducted using a potentiostat (Zive SP1, WonATech, Seoul, Republic of Korea) to investigate the as-fabricated working electrodes. In the three-electrode system, an Ag/AgCl electrode and platinum wire were employed as the reference and counter electrodes, respectively, and 1 M Na_2_SO_4_ was used as the electrolyte. The operating voltage ranges were set from −1.0 to 0 V, for the C-CA- and C-TL-based electrodes, and 0 to 1.0 V, for the C-CA@NiO- and C-TL@NiO-based electrodes. The current density for GCD measurements was set in the range from 1 to 10 A g^−1^. The specific capacitances ($C_{S}$, F g^−1^) of the single electrodes were obtained by evaluating the discharge energy densities (or charge energy densities, $E_{C}$) from the GCD curves using the integral of the discharging curves, according to the following equations [55]:

(2) $$E_{D}=\frac{I}{M}\int_{t\left(V_{\max}\right)}^{t\left(V_{\min}\right)}V\left(t\right)dt$$

(3) $$\;C_{S}=\frac{2\times E_{D}}{\Delta V^{2}}$$

where $E_{D}$ is the discharge energy density, I is the current, V is the voltage after the ohmic drop, ΔV is the operating voltage window, *M* is the mass of the active material, and t is the charge/discharge time. The coulombic efficiency (η) is then determined by the ratio of the discharging and charging energy densities, which is presented as follows [55].

(4) $$\eta =\frac{E_{D}}{E_{C}}$$

Moreover, the cell capacitance ($C_{\mathrm{Cell}}$) and coulombic efficiency of the ASC device were calculated using equations similar to those used in a three-electrode system obtained from the GCD curves, substituting the total mass of the active materials for the electrodes in the voltage range of 0–2.0 V. An EIS analysis of the ASC device was conducted under an applied alternating-current voltage of 10 mV in the frequency range of 10^−2^ to 10^5^ Hz. The energy density (E, Wh kg^−1^) and power density (P, kW kg^−1^) of the ASC device were calculated using the following relationships [56]:

(5) $$E=\frac{C_{\mathrm{Cell}}\times \Delta V^{2}}{2}\times \frac{1000}{3600}\;$$

(6) $$P=\frac{E}{\Delta t}\times \frac{3600}{1000}$$

where $C_{\mathrm{Cell}}$ from the GCD result is used.

## 3. Results and Discussion

### 3.1. Fabrication of the Al-Coated Current Collector, C-CA@NiO, and C-TL@NiO Materials

**Figure 1:**
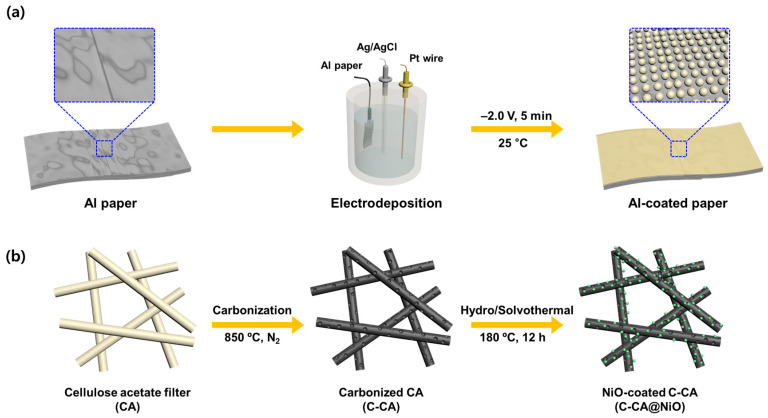
Schematic representation of (**a**) the synthesis of Al-coated paper through electrodeposition, and (**b**) the fabrication of NiO-coated C-CA (C-CA@NiO) materials via carbonization and hydro/solvothermal methods.

The process of preparing the materials for the electrodes and current collectors used in the supercapacitors is shown in Figure 1. Al paper retrieved from heated tobacco waste was utilized as the Al precursor material for electrodeposition. A uniform coating of Al onto another piece of Al paper filled the interstitial area, bridging the electrically disconnected regions. The surface of the Al-coated paper was coated with graphite to prevent degradation by an aqueous electrolyte [57]. In addition, CAs and TLs were collected from heated tobacco waste and carbonized for use as active materials in the supercapacitor electrodes. The NiO was then introduced onto the surfaces of C-CA and C-TL using a hydro/solvothermal method. The x (wt%) of NiO was 20.0 and 21.9 wt%, respectively. An ASC device was then assembled after comparing the electrochemical performance of the C-CA- and C-TL-based electrodes as the negative side and the C-CA@NiO- and C-TL@NiO-based electrodes as the positive side, selecting the appropriate electrodes.

**Figure 2:**
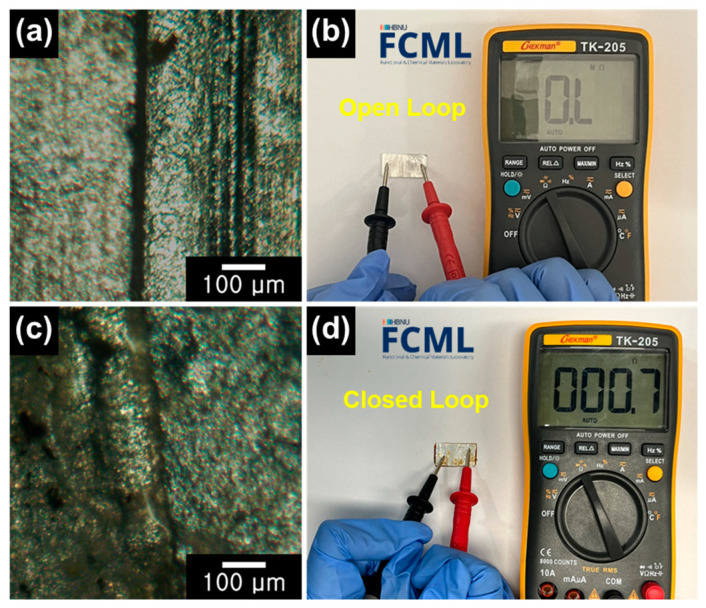
(**a**) OM image and (**b**) photograph of ohmmeter resistance measurement for pristine Al paper. (**c**) OM image and (**d**) photograph of resistance measurement for Al-coated paper.

Optical microscopy (OM) analysis was performed along with resistance measurements using an ohmmeter to observe the surface condition of the Al paper, as shown in Figure 2. The Al paper retrieved from the heated tobacco waste contained electrically disconnected areas, as confirmed by the black demarcations in the OM image (Figure 2a). The measured resistance between both ends of the Al paper obtained from the ohmmeter showed an overload (OL) value, confirming the nonconductive open-loop condition between the separated areas (Figure 2b). Figure 2c presents an OM image of the Al paper after the electrodeposition of Al metal. The image shows that the interstitial area is filled with conductive Al metal. Moreover, the ohmmeter resistance measurement between both ends showed a value of 0.7 Ω, signifying excellent electrical conductivity after electrodeposition (Figure 2d).

In general, electrodeposition is a method for coating a metal film onto a surface via an oxidation–reduction reaction [58]. The metal to be electrodeposited is connected to the anode, and the metal coated on the surface is connected to the cathode [59]. After the immersion of both metals in the electrolyte, a metal layer is deposited using an electrical source. Electrodeposition is regularly performed by applying a constant current using solvents such as aqueous solutions, organic solvents, and ionic liquids [60,61]. During the electrodeposition of Al, the interaction of an AlCl_3_ salt with organic solvents such as GBL yields AlCl_3_·RCl chloroaluminate [62]. As the concentration of AlCl_3_ increases, Al_3_Cl_10_^−^ ions are formed. Al metal is then coated onto the target object for electrodeposition through the reduction of Al_3_Cl_10_^−^ ions. As the concentration of AlCl_3_ increases, the development process of Al_3_Cl_10_^−^ ions (Equations (7)–(9)) and the electrodeposition process of Al metals from Al_3_Cl_10_^−^ precursors (Equation (10)) are represented as follows [63]:

AlCl_3_ + Cl^−^ → AlCl_4_^−^ (7)

AlCl_3_ + AlCl_4_^−^ → Al_2_Cl_7_^−^ (8)

AlCl_3_ + Al_2_Cl_7_^−^ → Al_3_Cl_10_^−^ (9)

2Al_3_Cl_10_^−^ + 3e^−^ → Al + 5AlCl_4_^−^ (10)

Hence, the Al metal was successfully electrodeposited onto Al paper for use as a highly conductive current collector for supercapacitor electrodes.

**Figure 3:**
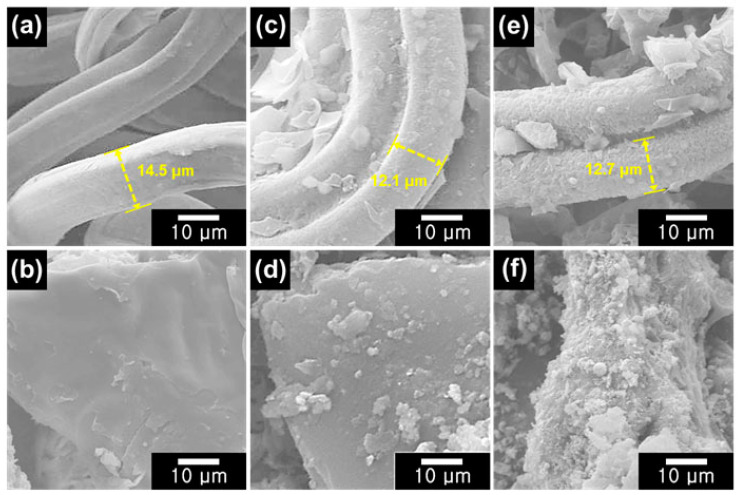
FE-SEM images of (**a**) CA, (**b**) TL, (**c**) C-CA, (**d**) C-TL, (**e**) C-CA@NiO, and (**f**) C-TL@NiO.

The morphological structures of the CA, TL, C-CA, C-TL, C-CA@NiO, and C-TL@NiO materials were investigated using field-emission scanning electron microscopy (FE-SEM) (Figure 3). Smooth surfaces were observed for pristine CA and TL materials (Figure 3a,b). It was clearly observed that the TL possessed bulk structures, without specific dimensions. On the other hand, CA was observed as a three-dimensional (3D) structure composed of fibers with diameters of ca. 14.5 μm. After the carbonization, the surface roughness of C-CA and C-TL increased compared with that of the pristine materials (Figure 3c,d). In the case of C-CA, the 3D structures were maintained with a reduced fiber size, with a thickness of ca. 12.1 μm. In addition, the surface roughness of C-CA@NiO and C-TL@NiO increased, confirming the successful incorporation of NiO into C-CA and C-TL using the hydro/solvothermal method (Figure 3e,f). The NiO particle diameter was measured as ca. 0.65 µm. In the case of C-CA@NiO, 3D structures were maintained, with fibers with an increased thickness of ca. 12.7 μm due to the successful incorporation of NiO.

The elemental compositions (C, O, and Ni) of the materials were analyzed using energy-dispersive X-ray spectroscopy (EDS), as shown in Table 1. The light elements C and O could not be easily detected by EDS; however, the ratio and atomic percentage of the two elements changed significantly following the carbonization and NiO incorporation [64]. Before carbonization, only C and O were detected in the CA and TL materials. In addition, TL exhibited a higher C/O ratio than CA. Despite the low C content in the precursor, the C elemental composition was higher in C-CA than in C-TL after carbonization. This phenomenon arises from the difference in the carbonization temperatures and conversion efficiencies of the organic constituents in CA and TL. Typically, for supercapacitor applications, most materials, including biomass waste, undergo carbonization within the temperature range of 800–900 °C [9,65]. Simple CA, predominantly featuring β-1,4-glycosidic bonds, transitions into carbon within a lower carbonization temperature range of 200 to 350 °C [66,67]. However, TL is primarily composed of lignin, which contains complex bonds and functional groups such as aromatic rings, hydroxyl groups, and carboxyl groups [68,69,70]. This complexity necessitates an optimal carbonization temperature nearing 900 °C for the decomposition of these diverse functional groups [71]. As a result, the chemically simplistic C-CA exhibited a higher C content than the structurally intricate C-TL. C, O, and Ni were detected in C-CA@NiO and C-TL@NiO, indicating the successful incorporation of NiO and the retained carbonized state of the materials.

**Figure 4:**
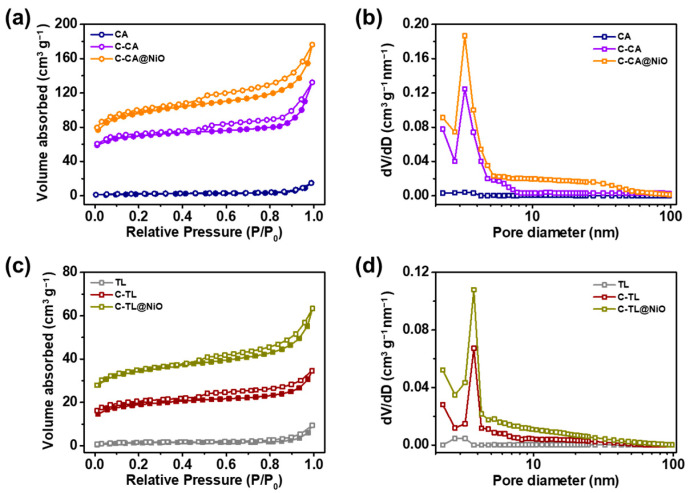
(**a**) N_2_-sorption isotherms and (**b**) BJH pore distribution curves of CA, C-CA, and C-CA@NiO. (**c**) N_2_-sorption isotherms and (**d**) BJH pore distribution curves of TL, C-TL, and C-TL@NiO.

N_2_-sorption isotherms and the associated Brunauer–Emmett–Teller (BET) surface area and the Barrett–Joyner–Halenda (BJH) pore distributions were investigated to assess the porous structure of the CA- and TL-based materials (Figure 4). For pristine CA and TL, typical type-II hysteresis curves were observed, indicating a nonporous nature [72]. However, type IV hysteresis loops were observed for C-CA, C-TL, C-CA@NiO, and C-TL@NiO materials, revealing their porous natures [73]. The BJH pore size distributions and BET-specific surface areas of the CA and TL materials are listed in Table 2. The pore sizes of the C-CA, C-TL, C-CA@NiO, and C-TL@NiO materials were determined to be 3.8, 3.3, 3.7, and 3.2 nm, respectively. The surface areas of CA, C-CA, and C-CA@NiO were 5.9, 275, and 360 m^2^ g^−1^, respectively. The surface areas of the CA materials increased significantly after carbonization and the incorporation of NiO. These increases in the surface area were ascribed to the simple structure of the CA materials, which allowed for the formation of mesopores during carbonization through the release of CO_2_ and H_2_O vapors from the decomposed functional groups [74]. Further increases in the BET surface area were observed with NiO incorporation owing to the formation of 0.65 μm particles on the surface of the carbonized C-CA. The BET surface areas of TL, C-TL, and C-TL@NiO were calculated to be 5.1, 65, and 118 m^2^ g^−1^, respectively. The surface area of C-TL marginally increased owing to the complex functional groups of TL, leading to limited pore formation and surface area increase [75]. Therefore, the CA-based materials exhibited large increases in surface area, suggesting their potential efficacy as active materials for supercapacitor applications in terms of improved contact area and charge storage.

X-ray diffraction (XRD) patterns were obtained to identify the crystal structures of C-CA, C-TL, C-CA@NiO, and C-TL@NiO (Figure S1). For C-CA and C-TL, the diffraction peaks at 22.9° corresponded to the (002) plane, indicating a disordered carbonaceous peak [76]. Additionally, the peak intensity of C-CA was higher than that of C-TL, indicating a higher proportion of carbon. In addition, C-CA@NiO and C-TL@NiO exhibited characteristic peaks of NiO. Specifically, the diffraction peaks for C-CA@NiO and C-TL@NiO were detected at 37.3, 43.3, 62.8, 75.4, and 79.3°, corresponding to the (111), (200), (220), (311), and (222) planes of the face-centered cubic NiO (JCPDS Card No.78-0643), respectively [77]. Moreover, thermogravimetric analysis (TGA) was carried out in the temperature range from 40 to 850 °C under N_2_ atmosphere, with a heating rate of 5 °C min^−1^ (Figure S2). After combustion was completed, the remaining C-CA, C-TL, C-CA@NiO, and C-TL@NiO material weight percentages were determined to be 81.1, 78.7, 69.4, and 69.2 wt%, respectively. It was clear that the C-CA and C-TL materials showed sharp mass losses near 100 °C due to the evaporation of moisture. Then, a gradual decrease in weight losses continued for C-CA and C-TL up to the final temperature of 850 °C owing to the thermal stability of carbon materials [78,79]. For C-CA@NiO and C-TL@NiO, the decrease in weight losses were suppressed compared with those of the precursors C-CA and C-TL owing to the incorporation of NiO. Since NiO materials were covered and incorporated into carbons, the thermal stability of the C-CA@NiO and C-TL@NiO materials was further increased up to near 600 °C. However, sudden decreases in weight losses were observed for both materials in the temperature range of 600 to 700 °C. This sudden weight loss may be due to the catalytic effect caused by the NiO, which lowered the decomposition temperature of the carbonaceous C-CA and C-TL materials [80,81]. Therefore, XRD and TGA results indicated the successful incorporation of NiO into the C-CA and C-TL materials.

### 3.2. Electrochemical Properties of C-CA@NiO- and C-TL@NiO-Based Electrodes

**Figure 5:**
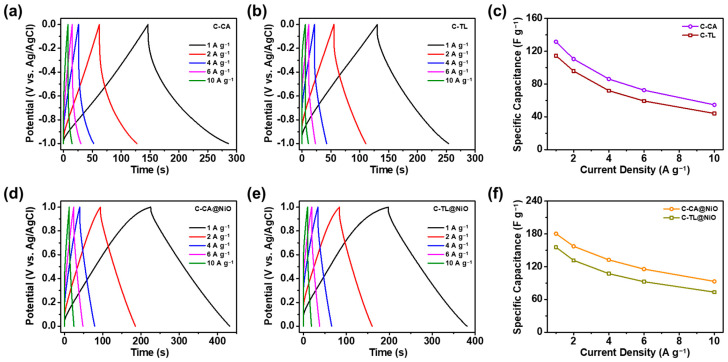
Galvanostatic charge-discharge (GCD) curves of (**a**) C-CA- and (**b**) C-TL-based electrodes at various current densities ranging from 1 to 10 A g^−1^. (**c**) Specific capacitances of C-CA- and C-TL-based electrodes computed from GCD results. GCD curves of (**d**) C-CA@NiO- and (**e**) C-TL@NiO-based electrodes at different current densities. (**f**) Specific capacitances of C-CA@NiO- and C-TL@NiO-based electrodes obtained from GCD results.

GCD analysis was conducted to investigate the electrochemical performance of the C-CA-, C-TL-, C-CA@NiO-, and C-TL@NiO-based electrodes in a three-electrode system, with 1 M Na_2_SO_4_ solution employed as an electrolyte. The GCD curves of the C-CA- and C-TL-based electrodes were evaluated over a potential range of −1.0–0 V at different current densities from 1 to 10 A g^−1^ [82]. The recorded curves are shown in Figure 5a,b. Both curves affirm their superior capacitive qualities. The C-CA-based electrodes exhibited specific capacitances of 131.6, 110.5, 91.7, 77.5, and 60.1 F g^−1^, and the C-TL-based electrodes exhibited values of 114.6, 95.8, 71.9, 59.3, and 44.1 F g^−1^ at current densities of 1, 2, 4, 6, and 10 A g^−1^, respectively. The C-CA- and C-TL-based electrodes preserved 41.5 and 38.5% of the specific capacitances, respectively (relative to the specific capacitances at 1 A g^−1^), even at a high current density of 10 A g^−1^; this demonstrates the high rate of capability of electrodes based on Al-coated current collectors. In addition, the coulombic efficiencies of both electrodes were calculated to evaluate their energy-storage abilities by computing the ratio of the discharge and charge energy densities [83]. In the case of the C-CA-based electrode, the charge and discharge energy densities were 270.7 and 236.9 mWh g^−1^ at 1 A g^−1^, respectively, and the coulombic efficiency was calculated as 87.5%. The C-TL-based electrode exhibited a coulombic efficiency of 85.2%, with charge and discharge energy densities of 242.1 and 206.3 mWh g^−1^, respectively. Given their coulombic efficiencies, both electrodes were suitable as negative electrodes; however, the C-CA-based electrode exhibited superior electrochemical performance compared to the C-TL-based electrode. This is attributed to the 3D fibrous structures of C-CA, promoting the development of porous carbon formations. The porous structure exhibited a high surface area, providing favorable conditions for the movement and storage of electrons, which enhanced the electrochemical performance of the C-CA materials [84]. The specific capacitances of the C-CA- and C-TL-based electrodes, as inferred from the GCD curves, are shown in Figure 5c.

The GCD curves for the C-CA@NiO- and C-TL@NiO-based electrodes were obtained to verify the compatibility of the NiO-coated carbon materials as positive electrodes in the potential range of 0 to 1.0 V at different current densities, as shown in Figure 5d,e [85]. The specific capacitances of the C-CA@NiO-based electrode were 180.4, 157.3, 132.5, 115.6, and 93.1 F g^−1^, and those of the C-TL@NiO-based electrode were 155.5, 131.6, 107.2, 92.5, and 73.4 F g^−1^ at current densities of 1, 2, 4, 6, and 10 A g^−1^, respectively (Figure 5f). Similar to the comparative results from the C-CA- and C-TL-based electrodes, the specific capacitance of the C-CA@NiO-based electrode was higher than that of the C-TL@NiO-based electrode, owing to the porous characteristics generated by the fibril structures in the CAs [84]. The C-CA@NiO- and C-TL@NiO-based electrodes exhibited rate capabilities of 51.6 and 47.2%, respectively, at 10 A g^−1^, confirming the enhanced ion diffusion following the incorporation of NiO, which increased the surface area [86]. In addition, the coulombic efficiencies of the C-CA@NiO- and C-TL@NiO-based electrodes were calculated to be 90.3 and 88.4%, respectively, verifying the suitability of the materials for supercapacitor applications. Similar to the previously obtained results, the C-CA@NiO-based electrodes exhibited better electrochemical performance than did the C-TL@NiO-based electrodes. Thus, the C-CA- and C-CA@NiO-based electrodes exhibited superior electrochemical performance compared to that of the C-TL- and C-TL@NiO-based electrodes in the potential ranges of −1.0 to 0 V and 0 to 1.0 V for application in ASC devices.

### 3.3. Electrochemical Performance of the Assembled ASC Device

From electrochemical analysis using a three-electrode system, the C-CA-based electrode exhibited superior performance compared to the C-TL-based electrode in the potential range of −1.0 to 0 V. Similarly, the C-CA@NiO-based electrode demonstrated better performance than the C-TL@NiO-based electrode in the range of 0 to 1.0 V. By employing C-CA- and C-CA@NiO-based electrodes with different operating potentials, the voltage window can be extended to provide positive and negative electrodes for the ASC device [87]. Therefore, a solid-state ASC (C-CA@NiO//C-CA) was assembled using C-CA@NiO as the positive electrode, C-CA as the negative electrode, and PVA/Na_2_SO_4_ as the gel electrolyte. Generally, the charges in the positive and negative electrodes must be equal to minimize the capacitance loss for supercapacitor performance ($Q_{+}=Q_{-}$) [88]. The amount of charge stored in each electrode can be calculated by multiplying the measured specific capacitance, the operating potential window, and the mass of the active material (Q=C×ΔV×M) [89]. By combining these two equations, the gravimetric ratios of the active materials in the negative and positive electrodes were calculated according to the following equation [90]:

(11) $$\frac{M_{-}}{M_{+}}=\frac{C_{+}\times \Delta V_{+}}{C_{-}\times \Delta V_{-}}.$$

The gravimetric ratios of C-CA and C-CA@NiO were calculated from the GCD results obtained at various current densities. The ratios calculated from the GCD results were 1.37, 1.42, 1.44, 1.49, and 1.55 at current densities of 1, 2, 4, 6, and 10 A g^−1^, respectively. Based on the determined values, the optimal ratio of the ASC device was determined by calculating the average of the 5 values, which was 1.45. Therefore, the ASC device was prepared by assembling C-CA@NiO- and C-CA-based electrodes while balancing the charge ratio.

**Figure 6:**
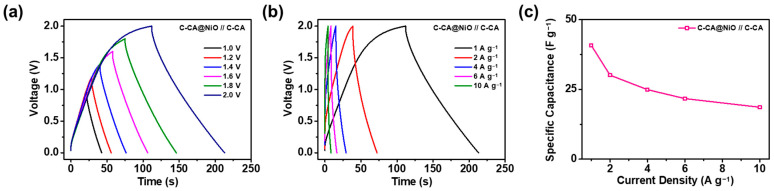
GCD curves of the as-prepared C-CA@NiO//C-CA ASC device in the study with (**a**) different voltage ranges and (**b**) current densities. (**c**) Specific capacitances of the ASC device computed from the GCD curves within the 2.0 V voltage range.

The electrochemical performance of the as-prepared C-CA@NiO//C-CA ASC device was examined to confirm its suitability as a practical energy storage device. Figure 6a shows the GCD curves of the ASC device at a 1 A g^−1^ current density, extending to voltages of 2.0 V. The ASC device exhibited stable behavior up to 2.0 V, without performance degradation, owing to the increased surface area and ability to store charges through the incorporation of NiO into the C-CA materials. The specific capacitances measured from the GCD curves were 18.7, 20.4, 24.0, 27.5, 33.6, and 40.8 F g^−1^ in the operating voltage windows of 1.0, 1.2, 1.4, 1.6, 1.8, and 2.0 V, respectively. The specific capacitance increases with the broadening of the operating voltage window owing to the pseudocapacitive reaction [24]. Moreover, the coulombic efficiencies were calculated as 89.3, 87.1, 88.2, 86.9, 88.6, and 87.5%, respectively, indicating the reversible properties of the ASC device, even at a high voltage of 2.0 V. Figure 6b presents the GCD curves of the ASC device at different current densities within the 2.0 V voltage window. The ASC device exhibited specific capacitances of 40.8, 30.1, 24.9, 21.7, and 18.6 F g^−1^, with coulombic efficiencies of 87.5, 86.8, 85.6, 84.3, and 83.8% at current densities of 1, 2, 4, 6, and 10 A g^−1^. Accordingly, the as-prepared ASC device assembled using the C-CA@NiO- and C-CA-based electrodes, with an Al-coated current collector, exhibited consistent electrochemical performance, as confirmed by the GCD curves. Figure 6c shows the plot of the specific capacitances for the ASC device evaluated as a function of the current density.

**Figure 7:**
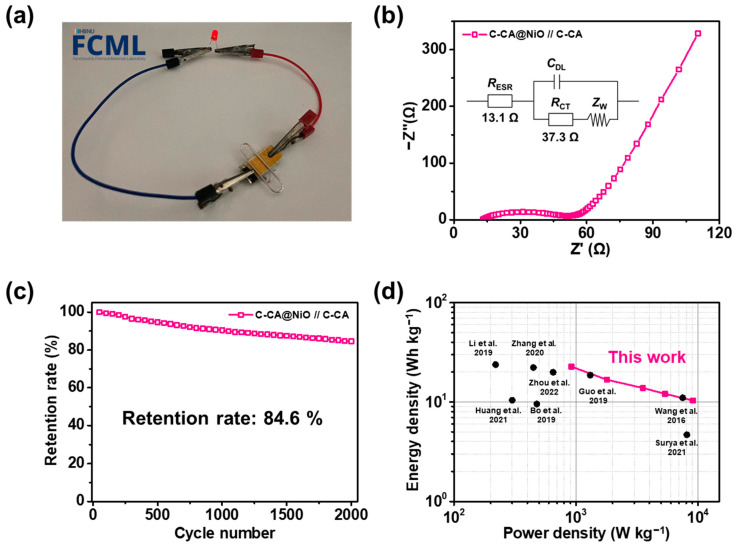
(**a**) Digital photograph of the C-CA@NiO//C-CA ASC device illuminating a red LED. (**b**) EIS analysis of the ASC device. (**c**) Long-term cycling test of the ASC device. (**d**) Ragone plot of the ASC device compared to various recycled biomass waste supercapacitor devices. Reproduced by: Li et al., 2019 [96], Zhang et al., 2020 [97], Zhou et al., 2022 [98], Guo et al., 2019 [99], Wang et al., 2016 [100], Huang et al., 2021 [101], Bo et al., 2019 [102], and Surya et al., 2021 [103].

Figure 7a presents a digital photograph of the prepared C-CA@NiO//C-CA ASC device. The active materials of the positive and negative electrodes were successfully applied to an Al-coated current collector. A gel-type electrolyte was fabricated by dissolving 1 M Na_2_SO_4_ in a 10.0 wt% PVA solution, which was then applied between the electrodes. The operating voltage window of the fabricated ASC device widened to 2.0 V, sufficient to power a 1.8 V red LED. The device was fixed using a polyimide film and a clip to minimize the contact resistance at the electrode–electrolyte interface and to maintain the ion-transport abilities, which were evaluated using EIS (Figure 7b). In addition, an equivalent circuit diagram corresponding to the ASC device is shown in the inset, characterized by the equivalent series resistance *R*_ESR_, charge-transfer resistance *R*_CT_, Warburg impedance *Z*_W_, and double-layer capacitance *C*_DL_ [91]. *R*_ESR_ refers to the total resistance, including the electrode, ionic, current collector, and contact resistances, which can be evaluated in the high-frequency region [92]. *R*_CT_ includes the faradaic resistance at the electrode–electrolyte interface [93]. The *R*_ESR_ value determined from the EIS plot was 13.1 Ω, which is related to the high conductivity of the active materials. However, a relatively high *R*_CT_ value of 37.3 Ω was measured due to the slight decomposition of the electrolyte during charge/discharge cycling and the contact resistance resulting from the use of Al-coated current collectors [94,95]. In the low-frequency region, a straight line with a high slope was evident, attributable to Warburg diffusion, indicating the excellent capacitive behavior of the ASC device [91]. In addition, a cyclability test of the device was performed to ensure its long-term stability over 2000 cycles at a fixed current density of 1 A g^−1^ (Figure 7c). The C-CA@NiO//C-CA ASC device exhibited a retention rate of 84.6% of its initial performance, confirming the stability of the ASC device which employed active materials and Al-coated current collectors sourced from heated tobacco waste. Finally, the energy and power densities of the ASC device were computed and compared with those of other devices using various types of biomass wastes, as shown in Figure 7d [96,97,98,99,100,101,102,103]. The C-CA@NiO//C-CA ASC device had a maximum energy density of 22.7 Wh kg^−1^, with a power density of 0.92 kW kg^−1^ at 1 A g^−1^. However, when the current density changed to 10 A g^−1^, the power density increased to 9.07 kW kg^−1^, whereas the energy density decreased to 10.3 Wh kg^−1^. Compared with the devices from other studies, the ASC device exhibited high energy and power densities, demonstrating the excellent electrochemical performance of the device, which employed Al-coated current collectors and active materials obtained from heated tobacco waste. Thus, the C-CA@NiO//C-CA ASC device using Al-coated current collectors emerges as an innovative energy storage device.

## 4. Conclusions

This study demonstrated the potential to recycle the components of heated tobacco waste as current collectors and active materials for supercapacitor electrodes. The Al paper from heated tobacco waste was used as the Al precursor. The electrodeposition of Al onto another Al paper filled the discontinuous areas and electrically connected both ends. The resulting Al-coated paper served as a highly conductive current collector. In addition, CA filters and TL were carbonized under an N_2_ atmosphere (C-CA and C-TL), followed by integration with nickel oxide (C-CA@NiO and C-TL@NiO) via the hydro/solvothermal method, providing active materials for the electrodes. All electrodes were prepared using Al-coated current collectors. Compared to the C-TL- and C-TL@NiO-based electrodes, the C-CA- and C-CA@NiO-based electrodes exhibited ca. 1.15 and 1.16 times higher specific capacitance at a current density of 1 A g^−1^. This enhancement in specific capacitance was attributed to the inherently high porosity of C-CA and the additional pseudocapacitance provided by C-CA@NiO. For practical application, the ASC device was assembled using C-CA@NiO as the positive electrode and C-CA as the negative electrode. The device demonstrated an exceptional specific capacitance of 40.8 F g^−1^ at 1 A g^−1^, with a widened voltage window of 2.0 V. Moreover, the ASC device exhibited an acceptable retention rate of 84.6% of its initial value after 2000 cycles. The specific capacitance and cyclability of the ASC device were attributed to the successful electrodeposition of Al, which facilitated electrical connectivity and increased the porosity and pseudocapacitance of the C-CA@NiO material. Thus, the successful recycling of heated tobacco waste into high-performance energy storage devices represents a new avenue for the fabrication of ecofriendly supercapacitor devices.

## Figures and Tables

**Table 1:** Elemental compositions of various CA- and TL-based materials fabricated in this study *^a^*.

Sample	Element (Atomic %)			C/O Ratio
	C	O	Ni	
CA	54.8	45.2	-	1.2
TL	61.4	38.6	-	1.6
C-CA	85.5	14.5	-	5.9
C-TL	79.2	20.8	-	3.8
C-CA@NiO	78.2	14.0	7.8	5.6
C-TL@NiO	74.6	17.9	7.5	4.2

*^a^* Elemental compositions of the samples were obtained using the EDS mode within the FE-SEM setup (beam current: 10.0 μA; accelerating voltage: 10.0 kV).

**Table 2:** Brunauer–Emmett–Teller (BET) specific surface areas and pore volumes of CA- and TL-based materials.

Samples	BET Specific Surface Area (m^2^ g^−1^) *^a^*	Pore Size (nm)	Pore Volume (cm^3^ g^−1^) *^b^*
CA	5.9	-	0.016
TL	5.1	-	0.013
C-CA	275	3.8	0.088
C-TL	65	3.3	0.029
C-CA@NiO	360	3.7	0.105
C-TL@NiO	118	3.2	0.057

*^a^* Calculated using the BET method. *^b^* Total pore volume.

## Data Availability

Data are contained within the article.

## Supplementary Materials

The following supporting information can be downloaded at: https://www.mdpi.com/article/10.3390/ma16196454/s1, Figure S1: X-ray diffraction (XRD) spectra of C-CA, C-TL, C-CA@NiO, and C-TL@NiO materials. Figure S2: TGA thermograms of C-CA, C-TL, C-CA@NiO, and C-TL@NiO materials.
