# Supplementary material for: Preparation of a High-Performance Asymmetric Supercapacitor by Recycling Aluminum Paper and Filter Components of Heated Tobacco
Source: Materials (Basel). 2023 Sep 28;16(19):6454. doi: 10.3390/ma16196454 (PMC10573335; doi:10.3390/ma16196454)
Supplement: Supplementary file 1 [file materials-16-06454-s001.zip › materials-2585689-supplementary.pdf]

materials

Supplementary Materials

Preparation of a High-Performance Asymmetric Supercapacitor by Recycling Aluminum Paper and Filter Components of Heated Tobacco

Ha-Yeong Kim [1,†], Suk Jekal [1,†], Chan-Gyo Kim [1], Jungchul Noh [2], Jiwon Kim [1], Yeon-Ryong Chu [1], Zambaga Otgonbayar [1], Won-Chun Oh [3], Sang Hun Lee [1] and Chang-Min Yoon [1,*]

[1] Department of Chemical and Biological Engineering, Hanbat National University, Daejeon 34158, Republic of Korea
[2] McKetta Department of Chemical Engineering and Texas Material Institute, The University of Texas at Austin, Austin, TX 78712, USA
[3] Department of Advanced Materials Science and Engineering, Hanseo University, Seosan-si 31962, Republic of Korea
* Correspondence: cmyoon4321@hanbat.ac.kr; Tel.: +82-42-821-1528; Fax: +82-42-821-1593
† These authors contributed equally to this work.

Citation: Kim, H.-Y.; Jekal, S.; Kim, C.-G.; Noh, J.; Kim, J.; Chu, Y.-R.; Otgonbayar, Z.; Oh, W.-C.; Lee, S.H.; Yoon, C.-M. Preparation of a High-Performance Asymmetric Supercapacitor by Recycling Aluminum Paper and Filter Components of Heated Tobacco. *Materials* **2023**, *16*, x. https://doi.org/10.3390/xxxxx

Academic Editors: Stefano Guarino and Flaviana Tagliaferri

Received: 15 August 2023
Revised: 25 September 2023
Accepted: 26 September 2023
Published: 28 September 2023

Copyright: © 2023 by the authors. Submitted for possible open access publication under the terms and conditions of the Creative Commons Attribution (CC BY) license (https://creativecommons.org/licenses/by/4.0/).

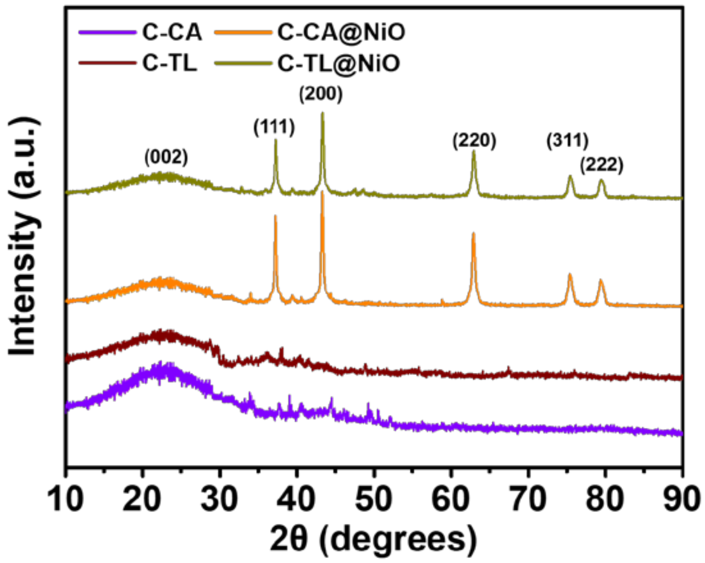

Figure S1. X-ray diffraction (XRD) spectra of C-CA, C-TL, C-CA@NiO, and C-TL@NiO materials.

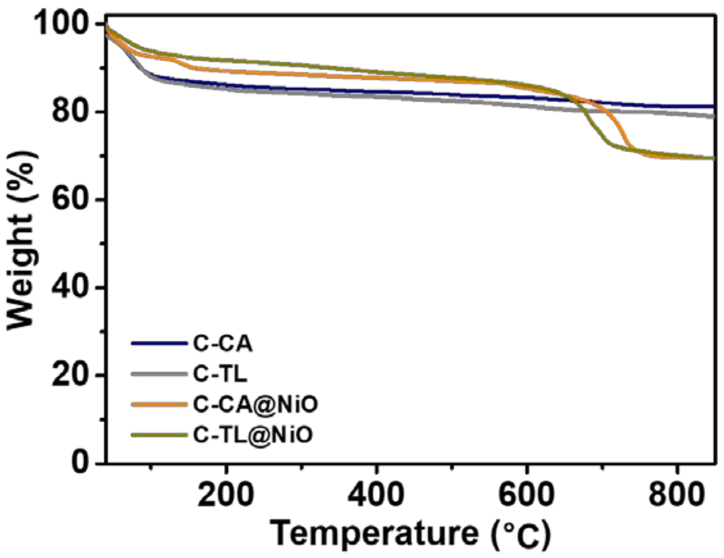

Figure S2. TGA thermograms of C-CA, C-TL, C-CA@NiO, and C-TL@NiO materials.
